# Risk Factors for the Development of Malignancies Post-Transplantation in Kidney Transplant Recipients

**DOI:** 10.3390/biomedicines13102346

**Published:** 2025-09-25

**Authors:** Kalliopi Vallianou, Ioannis Bellos, Vassilis Filiopoulos, Chrysanthi Skalioti, Pagona Lagiou, Vassiliki Benetou, Smaragdi Marinaki

**Affiliations:** 1 Clinic of Nephrology and Renal Transplantation, Medical School, National and Kapodistrian University of Athens, Laiko Hospital, 11527 Athens, Greece; bellosg@windowslive.com (I.B.); vassilis.filiopoulos@hotmail.com (V.F.); c_skalioti@yahoo.com (C.S.); smaragdimarinaki@yahoo.com (S.M.); 2 Department of Hygiene, Epidemiology and Medical Statistics, Medical School, National and Kapodistrian University of Athens, 11527 Athens, Greece; pdlagiou@med.uoa.gr (P.L.); vbenetou@med.uoa.gr (V.B.)

**Keywords:** kidney transplantation, malignancy, long-term outcomes, immunosuppression

## Abstract

**Background/Objectives**: Malignancies constitute a major cause of death among kidney transplant recipients, and their incidence is increasing globally. We aimed to estimate the frequency of de novo malignancies and identify factors associated with their occurrence among kidney transplant recipients. **Methods**: Data were derived from the medical records of patients who received a kidney transplant between January 1979 and December 2023 in “Laiko” University Hospital in Athens, Greece. Κidney transplant recipients with a diagnosis of de novo malignancy were compared with recipients without malignancy and were matched for age, sex and year of transplantation. Demographic and clinical characteristics, data on immunosuppression and cancer type were recorded. Multivariate logistic regression was employed to identify possible risk factors for cancer occurrence. **Results**: Out of 2986 recipients, 268 (8.98%) developed malignancies within a median time of 8 (interquartile range—IQR: 4–16) years after transplantation. Of them, 59.3% were males, the median age at transplantation was 48 (IQR: 39–57) years and the median dialysis vintage was 31.5 (IQR: 9.5–70) months. In addition, 17.2% had a history of rejection. The majority (66.7%) received a combination of mycophenolate and a calcineurin inhibitor with or without steroids. The most frequent malignancies were lung cancer (13%) and post-transplant lymphoproliferative disease (13%), followed by Kaposi sarcoma (8.2%). At diagnosis, 37% had generalized end-stage disease and 19% had aggressive disease with poor prognosis. In multivariate analysis, a history of rejection (odds ratio—OR = 1.75, 95% CI = 1.04–2.94) and glomerulonephritis as primary kidney disease (OR = 2.23, 95% CI = 1.06–4.67) were both significantly associated with malignancy development, whereas immunosuppressive medication was not. **Conclusions:** Cancer occurrence among kidney transplant recipients was related to the cumulative burden of immunosuppression rather than a specific immunosuppressant.

## 1. Introduction

Long-term outcomes of kidney transplantation have greatly improved during the last decades, with 10-year graft survival reaching 77% [1,2]. Death with functioning graft constitutes a major cause of graft loss, and 18.8–22.1% of kidney transplant recipients die with a functioning graft [3,4]. Cardiovascular disease (CVD) is traditionally the leading cause of death, though CVD is occurring with decreasing frequency as a result of the advances in prevention and treatment. On the other hand, malignancies have emerged as a major cause of morbidity and mortality and account for up to 27% of deaths [3,5]. In some cases, cancer is the leading cause of death among kidney transplant recipients [6,7].

Indeed, the cumulative incidence of de novo malignancies following kidney transplantation is 4–5% after five years, 10% after 10 years and 25% after 20 years [8,9]. The standardized incidence ratio (SIR) of de novo cancer in kidney transplant recipients compared with the general population is 2.1–3.9 [10]. The risk is markedly increased for many cancers (e.g., non-melanoma skin cancer (NMSC), lip, anal/perineum, Kaposi sarcoma, post-transplant lymphoproliferative disorder (PTLD), kidney, thyroid), mildly increased for others (e.g., lung, liver, stomach, colon, melanoma, cervical) and not particularly increased for some (breast, prostate, pancreas, ovarian, brain) [8,11,12,13,14].

The pathogenesis of cancer after transplantation is multifactorial. Apart from the traditional risk factors, such as age, sex, smoking and sun exposure, factors related to kidney disease and immunosuppression seem to play an important role [9,15]. The uremic environment in chronic kidney disease is believed to affect carcinogenesis through systemic inflammation and altered immune function, while acquired cystic kidney disease (ACKD) is strongly associated with renal cell carcinoma [9,16,17]. The most crucial contributor to cancer development is related to the effect of immunosuppressive treatment and the cumulative burden of immunosuppression in particular. Mechanisms implicated include decreased DNA repair, impaired immunosurveillance against malignant cells and viruses, increased angiogenesis and the direct oncogenic effects of immunosuppressants [9,15]. In the context of reduced immunosurveillance, oncogenic viruses have enhanced action through primary infection or reactivation and are closely associated with cancer growth, such as Epstein–Barr Virus (EBV) with PTLD and Human Herpesvirus 8 (HHV-8) with Kaposi sarcoma [9,15].

Most epidemiological data on de novo malignancy after kidney transplantation derive from North America and Northern and Western Europe [11,12,18,19,20,21]. Few data from Italy and Spain are available [22,23]. In Greece, cancer incidence tends to be lower than Europe on average and cancer epidemiology is affected by specific factors such as a Mediterranean diet, low alcohol consumption, increased sun exposure and high smoking prevalence [24]. On the other hand, Greece falls behind in cancer screening and prevention strategies, and while living donation rates are high, time on the waiting list for a deceased kidney transplant can be up to 9 years, prolonging the effect of chronic kidney disease for many dialysis patients [24,25]. However, malignancies have not been previously studied in the kidney transplant population in Greece. Thus, the aim of the study was to record cancer incidence and investigate the potential risk factors associated with its development in a large cohort of kidney transplant recipients followed at a tertiary university hospital in Athens, Greece, over a period of almost 45 years. The role of immunosuppressive regimens and distinct immunosuppressive drugs in cancer development was investigated in particular.

## 2. Materials and Methods

We retrospectively collected data from the medical records of kidney transplant recipients followed at the Clinic of Nephrology and Renal Transplantation at Laiko General Hospital of Athens, Greece, between January 1979 and December 2023. The study was approved by the Institutional Review Board of the “Laiko” General Hospital (protocol number: 214, 20 January 2023) and is in accordance with the Declaration of Helsinki.

All kidney transplant recipients who developed de novo malignancy (either solid organ or hematologic malignancies) at least six months after transplantation were identified. Diagnosis was confirmed by histology. Patients with post-transplant recurrence of pre-existing cancer were excluded. Among NMSCs, we included those that required extensive surgical resection, radiation or chemotherapy. Patients with cancer were compared with a control group of kidney transplant recipients without cancer, selected from the initial reference population. The variables used for matching were sex, age at transplantation (±3 years) and year of transplantation (±1 year). A 1:1 matching strategy was employed, and the procedure was performed manually.

Demographic, clinical and immunological data on patients and controls, including body mass index (BMI), history of smoking, diabetes mellitus, primary kidney disease, dialysis vintage, number of transplantations, type of donor (living/deceased), viral infections (Hepatitis B Virus—HBV, Hepatitis C Virus—HCV, Cytomegalovirus—CMV, BK Virus—BKV), estimated glomerular filtration rate (eGFR) one year post-transplant, percentage of anti-HLA antibodies (human leucocyte antigen), presence of donor-specific antibodies (DSAs), HLA and ABO incompatibility were recorded. We also collected information on induction and maintenance immunosuppression medication and regimens, rejection occurrence, type and treatment. When there was more than one immunosuppressive regimen, the one administered at cancer diagnosis (or end of follow-up for controls) was chosen. Moreover, we identified cancer type, time from transplantation and eGFR at diagnosis. Follow-up was defined as the time from transplantation to cancer diagnosis for cases, and the time from transplantation to the last recorded clinic visit for controls. eGFR was calculated using the 2021 CKD-EPI (Chronic Kidney Disease-Epidemiology Collaboration) creatinine-based equation.

Regarding immunosuppressant administration, steroids were given as a bolus dose of 500 mg methylprednisolone (MP) during surgery, followed by 20–40 mg daily and slow tapering to 2–4 mg after 3–4 months. Three daily pulses of 500 mg^−1^ g were given as antirejection treatment, followed by gradual tapering. Azathioprine (Aza) was the first immunosuppressant used in transplantation along with steroids, and its dose was 2 mg/kg up to a maximum of 150 mg. Cyclosporine A (CsA) was introduced in the early 1980s, while tacrolimus (TAC) appeared in the mid-1990s. Maintenance CsA 2-h target levels were 500–600 mg/dL, and maintenance TAC trough levels were 5–7 ng/dL. Mycophenolate replaced azathioprine in immunosuppressive regimens starting in the late 1990s. Maintenance mycophenolate mofetil (MMF) dose was 1500–2000 mg daily (1080–1440 mg of mycophenolate sodium-MPA) when administered with CsA and 1000–1500 mg daily (720–1080 mg of MPA) when administered with TAC. Sirolimus was used as of 2000 and was replaced by everolimus in 2009. Target mTORi levels were 5–6 ng/mL when administered with MMF/MPA or only with steroids and 4–5 mg/mL when administered with a CNI. Antithymocyte globulin (ATG) replaced anti-CD3 antibody muromonab (OKT3) and was used for 7–14 days as induction or antirejection treatment at an initial dose of 1–1.5 mg/kg and later based on daily CD3+ lymphocyte count. Patients undergoing transplant before around 1998 received either no induction or induction with ATG/OKT3, whereas those undergoing transplant after 1998 received induction with either ATG/OKT3 or anti-IL2Ra Abs (antibodies against receptor a of interleukin 2-daclizumab or basiliximab).

### Statistical Analysis

Categorical variables are expressed as frequencies and percentages. Continuous variables following a normal distribution are expressed as means and standard deviation (SD), whereas skewed continuous variables are expressed as median and interquartile range (IQR). Differences were calculated using the McNemar test for categorical variables, Student’s paired *t*-test for normally distributed continuous variables and Wilcoxon test for skewed numerical variables. Statistical significance was defined as a *p*-value < 0.05.

Factors associated with cancer development were studied using logistic regression, with results expressed as odds ratios (ORs) and 95% confidence intervals (CIs). Initially, the role of separate immunosuppressants was evaluated, followed by simple logistic regression on malignancy occurrence and immunosuppressive regimens. We subsequently built a multiple regression model that included immunosuppressive regimens and major parameters associated with CKD and transplantation course: dialysis vintage, primary kidney disease, history of rejection and eGFR at one year post-transplant. Separate analyses were conducted on two patient subgroups. The first group included kidney transplant recipients with generalized/metastatic disease at diagnosis and aggressive disease with poor prognosis. The second group included patients who developed cancer types that are more frequently diagnosed among kidney transplant recipients, namely PTLD, Kaposi sarcoma, NMSC, kidney, thyroid and anal/perineum cancer. Finally, we performed a separate subgroup analysis stratified by transplant decade: 1979–1989, 1990–1999, 2000–2009 and 2010–2023. All statistical analyses were performed using STATA 14.2 (StataCorp LLC., College Station, TX, USA).

## 3. Results

### 3.1. Clinical Characteristics and Immunosuppressive Treatment of Kindey Transplant Recipients with and Without Malignancy

Between 1979 and 2023, 2986 kidney transplant recipients were followed at the specified clinic. Among them, 268 (8.98%) patients who developed de novo malignancy at least six months after transplantation were identified. The control group was chosen from the same population and was matched for sex, age at transplantation and year of transplantation. Table 1 depicts clinical characteristics and demographics of cases and controls.

Out of the 268 patients, 159 (59.3%) were males, whereas the median age at transplantation was 48 (IQR: 39–57) years in both groups. Dialysis vintage was similar in patients with cancer and controls (31.5 vs. 31 months). The most frequent primary kidney diseases were glomerulonephritis and polycystic kidney disease, while, notably, about in one third of patients in both groups the cause of nephropathy was unknown (36.2% vs. 35.4%). The majority received their first transplant and were not sensitized. Follow-up was longer for controls (8 vs. 13 years, *p* < 0.001). There were numerically more rejections in the cancer group (17.2% vs. 11.9%), though the difference between the two groups (*p* = 0.103) was not statistically significant. On the other hand, CMV infection was significantly more frequent in the control group (9.3% vs. 18.3%, *p* = 0.003).

Table 2 describes the immunosuppressive medications and regimens of cancer patients and controls, as induction, maintenance or antirejection treatment. About half of the patients in both groups (54.1% vs. 51.4%) received T cell depleting antibodies as induction, while one third (33.6% vs. 32.8%) received no induction. More than 90% of kidney transplant recipients were on a calcineurin inhibitor, and the most common regimen used (66.7% in both groups) was the combination of a calcineurin inhibitor and mycophenolate (mofetil of sodium) with or without steroids.

### 3.2. Characteristics of Kidney Transplant Recipients with Malignancies

Out of the 268 patients who developed cancer, 59.3% were men, with a median age at diagnosis of 58.5 (IQR: 50–66) years and median time from transplantation of 8 (IQR: 4–16) years. Median eGFR at diagnosis was 50 (IQR: 35–66) mL/min/1.73 m^2^.

**Figure 1:**
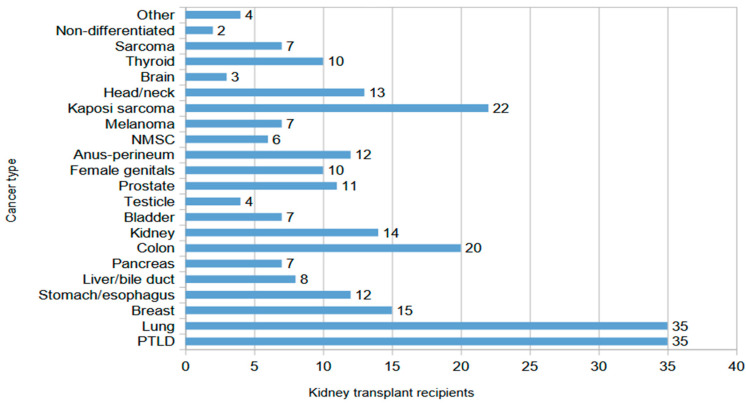
Cancer type and frequency in kidney transplant recipients (NMSC: non-melanoma skin cancer, PTLD: post-transplant lymphoproliferative disorder).

Figure 1 shows the cancer types and their frequency among the 268 patients with malignancies. Lung cancer and PTLDs were the most frequent malignancies (35 cases each—13%), followed by Kaposi sarcoma (22 cases—8.2%). Median time from transplantation to cancer diagnosis was 12 (IQR: 6–18.5), 7 (IQR: 3.5–9) and 1.5 (IQR: 1–3) years for PTLD, lung cancer and Kaposi sarcoma, respectively.

Other frequent malignancies were breast, colon and prostate cancer, and the common in CKD population, kidney and thyroid cancer. Interestingly, 37% of patients had generalized/metastatic disease on diagnosis, and 19% had aggressive disease with poor prognosis.

### 3.3. Risk Factors for Cancer After Kidney Transplantation

Malignancy occurrence was not associated with either separate immunosuppressants (CNI-CsA or TAC, MMF/MPA, Aza, mTORi, MP, MP pulses, ATG/OKT, Anti-IL2Ra AbsAs) or immunosuppressive regimens (ΜΜF/MPA+CNI±MP, Aza+CNI±MP, MMF/MPA+mTORi±MP, CNI+mTORi±MP, CNI+MP, Aza+MP). The kind of immunosuppression, induction, maintenance or antirejection was not related to risk of malignancy in the multiple regression model. However, a significant positive association was found with rejection history (OR = 1.75, 95% CI = 1.04–2.94) and glomerulonephritis as primary kidney disease (OR = 2.23, 95% CI = 1.06–4.67). Results are shown in Table 3.

### 3.4. Subgroup Analysis

We identified 99 (36.9%) kidney transplant recipients with generalized/metastatic disease on diagnosis and 51 (19%) with aggressive disease with poor prognosis, for a total of 150 out of 268 (55.9%) patients, who died or had short life expectancy because of the cancer. Multivariate analysis revealed no significant associations. However, an increased risk, though not significantly, was noted for patients with rejection history (OR = 1.76, 95% CI = 0.94–3.06) and glomerulonephritis as primary kidney disease (OR = 1.76, 95% CI = 0.9–3.32).

Secondly, we identified 99 (36.9%) patients with the cancer types that are more frequently diagnosed among kidney transplant recipients: PTLD, Kaposi sarcoma, NMSC, kidney, thyroid and anal/perineum cancer. No significant interactions were found. However, immunosuppressive treatment with MMF/MPA+mTORi±MP tended to show a protective effect on malignancy (OR = 0.35, 95% CI = 0.04–1.15, *p* = 0.073). Rejection history and primary kidney disease were not associated with increased cancer risk.

Finally, there were 40 (14.9%) patients transplanted between 1979 and 1989, 91 (33.9%) between 1990 and 1999, 85 (31.7%) between 2000 and 2009 and 52 (19.5%) between 2010 and 2023. The majority of kidney transplant recipients between 1979 and 1989 received long-term immunosuppression with Aza+CNI±MP (26/40 patients and 24/40 controls). Later, ΜΜF/MPA+CNI±MP was the main regimen, administered to 70/85 patients and 67/86 controls undergoing transplant between 2000 and 2009 and 48/52 patients and 44/50 controls between 2010 and 2023. Across all decades, the direction of associations remained consistent with the main analysis. No associations of induction treatment or immunosuppressive regimens and cancer development were noted. The risk for malignancy was higher in patients with rejection history (OR = 1.54, 95% CI = 0.46–5.17; OR = 1.69, 95% CI = 0.70–4.13; OR = 2.67, 95% CI = 0.79–9.03; OR = 1.95, 95% CI = 0.51–5.28 for the time groups), as well as in those with glomerulonephritis as primary kidney disease (OR = 2.82, 95% CI = 0.59–10.4; OR = 1.71, 95% CI = 0.41–7.26; OR = 2.25, 95% CI = 0.84–6.06; OR = 2.06, 95% CI = 0.57–8.52 for the groups). However, statistical significance was not reached in any individual group. A test for heterogeneity showed no effect modification (*p* = 0.735).

## 4. Discussion

We studied the frequency and risk factors associated with de novo malignancy post-transplant in a large cohort of kidney transplant recipients during a period of about 44 years in a specialized clinic of a University Hospital in Athens, Greece. From a total of 2986 kidney transplant recipients, 8.98% were diagnosed with a de novo malignancy, with PTLD, lung cancer and Kaposi sarcoma being the most frequent cancers. Among all factors studied, only history of graft rejection and glomerulonephritis as primary kidney disease were found to be associated with the development of malignancy.

Large epidemiological studies from the USA, the United Kingdom, Canada and Australia–New Zealand estimate cancer frequency to be between 5.2% and 12.1%, depending on the study design, follow-up period and whether NMSCs were included in the analysis [21,26,27,28,29]. Cumulative incidence of de novo malignancies has been found to increase with time after transplantation, starting from 4–5% after five years and gradually reaching 25% after 20 years [8,9]. In our cohort, the median time from transplantation to cancer diagnosis was 8 years, while almost 29% of the patients were diagnosed more than 15 years after transplantation. Most patients with early cancer (within the first two years post-transplant) were diagnosed with Kaposi sarcoma and PTLD. The first frequently occurs in the first two years post-transplant, and the second is known to have a biphasic distribution pattern with early (in the first two years) and late (after seven to ten years) occurrence [30].

The main factor that we aimed to investigate was immunosuppression. Calcineurin inhibitors are known to induce carcinogenesis by multiple mechanisms, among which are impaired DNA repair and increased TGF-β, VEGF and IL-6 production [11,31,32]. Studies have shown increased cancer risk when a CNI is added to the immunosuppressive regimen [33,34], as well as with higher target levels of CsA [35]. Azathioprine is related to microsatellite instability and DNA mutations and renders cells more vulnerable to oxidative damage from UV radiation [36,37,38]. Azathioprine is strongly associated with increased NMSC incidence [19,31,39] and with myelodysplastic syndrome and acute leukemia [36]. Mycophenolate mofetil/sodium blocks de novo purine synthesis, which is indispensable for lymphocytes and neoplastic cells [9,40]. As a result, besides the impaired immune function, this could suggest a protective mechanism against malignancy [19,41,42,43]. mTOR inhibitors block pathways leading to cell division and proliferation and induce apoptosis [44,45]. An anti-neoplastic effect has therefore been proposed. Studies have shown some protective effect, though this mainly affects NMSCs and Kaposi sarcoma [42,46,47]. The role of separate immunosuppressants used as induction, maintenance or rejection treatment was studied, but no significant association with malignancy risk was found. However, among our patients, the majority (94% vs. 92.3% in the cancer and control group) were on a CNI, about 73% on mycophenolate and a few (7.8% vs. 9%) on mTORis. Although the oncogenic effects of CNIs are well recognized, CNI-based regimens remain the first-line treatment due to their effectiveness in preventing graft rejection [31,32,34,48]. The widespread use of CNIs in the vast majority of patients and controls, combined with the relatively limited use of mTORis, may have obscured the potential harmful or protective effects of individual agents.

In addition to separate medications, immunosuppressive regimens were included in our analysis. Most patients (66.7%) were on MMF+CNI±MP, followed by Aza+CNI±MP (17.1 vs. 15.7 in the cancer and control group). In the main analysis, including all 268 patients, no association of a specific regimen with malignancy was found. The literature findings are not conclusive, but most studies show that the increased risk of cancer is present regardless of the regimen used [49,50,51]. An earlier study from Australia that included patients undergoing transplant between 1983 and 1986, reported similar malignancy occurrence with Aza+MP and CsA after a follow-up period of 20.6 years [51]. The ORION study compared Sir+TAC, Sir+MMF and TAC+MMF and found no difference in cancer after two years [50]. In pediatric kidney transplant recipients in Australia and New Zealand, the immunosuppressive regimen (TAC+MMF±MP, CsA+MMF±MP, CsA+Aza±MP or Aza+MP) was not associated with cancer incidence after a follow-up period of 13.4 years [49]. On the other hand, a prospective study showed that better medication adherence was associated with increased cancer incidence [52]. Beyond the specific effects of individual immunosuppressants on malignancy, other factors may contribute to cancer incidence, including the increased use of T-cell depleting therapies, higher rejection rates associated with less potent regimens and the elevated long-term CVD mortality associated with mTORis.

In the subgroup analysis including cancer types with highly increased frequency after transplantation, there was a trend towards a protective effect for patients receiving MMF/MPA+mTORi±MP. Although this analysis included only 99 patients and the result was not statistically significant, this trend could be explained by the absence of CNI and the presence of mTORi in the regimen. Cancers with significantly increased incidence post-transplant are a diverse group. However, CNIs seem to be implicated in the pathogenesis of some cancer types, like PTLD, Kaposi sarcoma and other virus-related malignancies [30,53]. On the contrary, there is some evidence that mTORis are related with a lower incidence of NMSCs and with the regression of Kaposi sarcoma lesions [47,54,55].

A history of rejection was associated with a 75% increased risk of de novo malignancy after transplantation, both in the main analysis and in the subgroup analysis of patients with very poor prognosis. Rejection type and treatment were not included in the regression analysis. However, the majority of the additional rejection episodes in the cancer group were T-cell-mediated (38/46 vs. 26/32 rejections in the cancer and control group). Steroid pulses were administered to a numerically greater number of patients with cancer compared to controls, whereas treatment with OKT3/ATG was similar between the two groups. Patients experiencing rejection were subsequently treated with higher doses of maintenance immunosuppression to suppress immune response and prevent recurrence. Similarly, the literature indicates that rejection episodes have been associated with malignancy regardless of the specific treatment, likely due to the higher cumulative immunosuppression administered to these patients both during and after the rejection episode [8,15,56]. Moreover, rejection is more frequently documented in high-risk patients, such as highly sensitized patients, undergoing an HLA-incompatible transplantation or receiving a second or third graft. These patients already received higher doses of immunosuppression even before the rejection diagnosis [8].

Interestingly, we did not find increased malignancy risk associated with T-cell depleting antibodies, namely for OKT3/muromonab and ATG/antithymocyte globulin, used either as an induction or rejection treatment. The depletion of CD4+ and CD8+ disrupts defense against malignant cells and viruses and leads to malfunction of other components of the immune system because of their regulatory role [8,57]. The association between ATG/OKT3 and malignancy is well established in the literature and is stronger, particularly with PTLD and with higher cumulative doses [9,29,43,58,59]. The approach used in our center is to minimize the cumulative ATG dose. A loading dose of 1–1.5 mg/kg and complementary doses based on daily CD3+ lymphocyte count, targeting 10–20 CD3+ lymphocytes for the shortest period needed, is administered, especially when given as induction.

Primary kidney disease is one of the most important CKD-related factors evaluated. We found that glomerulonephritis as primary kidney disease was a significant risk factor for de novo malignancy after transplantation, increasing risk by more than two times. This finding was constant when we checked for the subgroup with generalized/metastatic disease and poor prognosis. Glomerulonephritis could be primary or secondary to a systemic disease, such as vasculitis and lupus. Patients are usually treated with immunosuppressive medication for renal or extrarenal manifestations. Epidemiological data from the US and Australia–New Zealand do not reveal increased cancer incidence in kidney transplant recipients with glomerulonephritis [19,26]. However, a cohort study by Massicotte-Azarniouch and colleagues showed increased risk for patients with glomerulonephritis as primary kidney disease and history of immunosuppressive therapy before transplantation (HR 1.82, 95% CI 1.10–3.00) after a median follow-up of 5.7 years [60]. A recent retrospective cohort analysis from the UK found an increased cancer risk in patients with pre-existing glomerulonephritis (aHR 3.27, 95%CI: 1.10–9.77), without providing data on pre-transplant immunosuppression [61]. In our population, data on pre-transplant treatments were unavailable, preventing further analysis of potential associations. Nonetheless, since recipients with glomerulonephritis as the primary kidney disease do not receive different or intensified transplantation immunosuppression, the additional burden of immunosuppression before transplantation could account for their elevated risk of malignancy.

Viruses play an important role in malignancy development after transplantation. We recorded the data on BKV and CMV infection that were available in our population. BKV viruria has been associated with urothelial carcinoma [62]. In our study, BKV infection frequency did not differ between the cancer and control groups. Among the seven patients with ureteral/bladder cancer, only one (14.3%) had a history of BKV infection. We also noticed that CMV disease (primary infection or reactivation) was significantly more common in the control compared to the cancer group (18.3% vs. 9.3%, *p* = 0.003). This finding could be explained because, in the context of a CMV infection, immunosuppression is usually decreased not only during the infection but also afterwards, to avoid reactivation [63]. EBV involvement in the pathogenesis of PTLD is also known, with 75–90% of PTLD being EBV positive [30,43,64]. Out of the 35 patients with PTLD, there were available data on EBV for 17. Thirteen out of the 17 (76.5%) PTLD cases were EBV-related.

Year of transplantation is an important factor that can influence cancer occurrence as, over the course of 45 years, new immunosuppressants have been introduced and others withdrawn, and the general approach towards the transplant recipient has changed. Moreover, cancer screening techniques and detection sensitivity have improved over time [8,65]. Patient and graft survival have also increased, whereas cardiovascular mortality, particularly early post-transplant, responsible for up to 48% of deaths with a functioning graft in 1985–1995, has significantly decreased [4,5,8]. To address these issues, we matched patients and controls for year of transplantation (±1 year), as well as conducted a subgroup analysis by transplantation decade. Matching design helps account for temporal differences in clinical practice and diagnostic technology, and subgroup analysis showed that the trend of the associations observed in the main analysis remains consistent across decades. Although residual era-related differences cannot be ruled out, the combination of matching and subgroup analysis suggests that temporal changes are unlikely to have substantially influenced our findings.

The present study has both strengths and limitations. As far as we know, it is the first study to investigate de novo malignancy among kidney transplant recipients in Greece and includes a large number of patients. On top of that, it covers a long period of time, almost 45 years, contrary to most epidemiological studies in the literature, which do not exceed 20–25 years. Although it is a retrospective study, a methodological strength is the 1:1 matching by age, sex and year of transplantation, which minimizes the confounding associated with demographic factors and changes in clinical practice over time.

On the other hand, it is a single-center study, although the center is situated in Athens, the capital of Greece, covering the majority of citizens in Greece. Patient files are not fully digital. Consequently, only limited information was available for the total population of the 2986 kidney transplant recipients, which was insufficient for inclusion in the study. Critically, data on immunosuppression, rejection occurrence and treatments were not consistently available across the cohort. On top of that, due to the incomplete digitalization of patient records, matching could not be performed using a standardized statistical sampling method. Instead, it was performed manually by one author and verified by another, based on predefined criteria, potentially affecting objectivity and reproducibility. Data were collected from patients’ files starting in 1979; thus, information could be lost, damaged or misclassified, especially concerning cancer diagnosis, since the center or the patient file might not be updated on cancer occurrence. Data on immunosuppressive treatment administered prior to transplantation, history of malignancy prior to transplantation, viral infections such as EBV and cumulative dose of OKT3/ATG were not available. Parameters not included in the multivariate analysis, such as immunosuppressant levels/dose, rejection type and treatment, sensitization status, number of transplantations and viral infections could have an impact on the results. Additionally, the wide heterogeneity of cancer types—with varying pathogenesis and clinical behavior—complicates the interpretation of findings. The follow-up period was longer in the control group, which may introduce immortal time bias. The longer follow-up of controls could reflect better graft survival, potentially due to the lower immunological risk of recipients in need of a lower total dose of immunosuppression, or better patient survival, because of fewer comorbidities and lower mortality related to CVD and infections. In the earlier years, early acute rejection and graft loss rates were particularly high, and recipients with more compatible grafts that survived the first months post-transplant were more likely to be included in the control group. Finally, there could be residual confounding because of the observational design of the study.

## 5. Conclusions

In conclusion, this study contributes novel evidence by reporting, for the first time, information on de novo malignancies after kidney transplantation in Greece across an extended timeframe. We found that cancer frequency in Greece is comparable to that reported in North America and Northwestern Europe. Moreover, the study provides insights into potential risk factors, with a history of post-transplant rejection and glomerulonephritis as the primary kidney disease emerging as the main contributors to cancer development. On the other hand, no specific immunosuppressive agent or regimen was associated with an increased risk of malignancy, likely due to widespread use of CNIs and MMF/MPA in both patients and controls. The above suggest that the total dose of immunosuppression, rather that the specific regimen, may be related with cancer development.

Our findings may facilitate the identification of patients at a high risk for cancer, particularly those who have received higher cumulative doses of immunosuppression, including patients with a history of rejection and glomerulonephritis as the primary kidney disease. Patients should be educated on the importance of lifestyle modifications, including a healthy diet, smoking cessation and sun exposure minimization, as well as recommended cancer screening strategies, such as Pap cytology, mammography and colonoscopy. Follow-up should include monitoring adherence and providing reminders to ensure implementation of these preventive measures.

This study included a control group matched for age, sex and year of transplantation to strengthen its findings. However, the credibility of the results may be influenced by the retrospective, single-center study design, along with potential biases introduced by missing data and longer follow-up in controls. A prospective nationwide study would offer a more definitive assessment of the incidence and risk factors associated with de novo malignancy in kidney transplant recipients.

## Figures and Tables

**Table 1:** Demographic and clinical characteristics of kidney transplant recipients with malignancy and without malignancy.

	Kidney Transplant Recipients with Malignancy (Ν = 268)	Kidney Transplant Recipients Without Malignancy (Ν = 268)	*p*-Value
Sex (male)	159 (59.3)	159 (59.3)	1.000
Age at transplantation (years)	48 (39–57)	48 (39–57)	0.804
Dialysis vintage (months)	31.5 (9.5–70)	31 (15–72)	0.722
Primary kidney disease			0.392
● glomerulonephritis	72 (26.8)	61 (22.8)	
● polycystic kidney disease	43 (16.1)	50 (18.7)	
● hypoplastic kidneys/congenital nephropathy	19 (7.1)	26 (9.7)	
● obstructive nephropathy/lithiasis	20 (7.4)	13 (4.8)	
● diabetic nephropathy	7 (2.6)	11 (4.1)	
● hypertensive nephropathy	5 (1.9)	7 (2.6)	
● other	5 (1.9)	5 (1.9)	
● unknown	97 (36.2)	95 (35.4)	
Smoking (yes)	99 (36.9)	85 (31.7)	0.210
BMI (kg/m^2^)	23.6 (22–25.6)	23.9 (22–26)	0.210
HBV infection	18 (6.7)	18 (6.7)	0.441
HCV infection	23 (8.6)	29 (10.8)	0.720
Donor (living/deceased)	119/149 (44.4/55.6)	112/156 (41.8/58.2)	0.562
Transplant number			0.960
● 1st	259 (96.6)	251 (93.6)	
● 2nd	8 (3)	16 (6)	
● 3rd	1 (0.4)	1 (0.4)	
Transplant year			0.317
● 1979–1989	40 (14.9)	40 (14.9)	
● 1990–1999	91 (33.9)	92 (34.3)	
● 2000–2009	85 (31.7)	86 (32.1)	
● 2010–2019	50 (18.7)	48 (17.9)	
● 2020–2023	2 (0.8)	2 (0.8)	
PRAs (%)			0.548
● <5%	208 (77.6)	212 (79.1)	
● 5–70%	46 (17.2)	39 (14.5)	
● >70%	14 (5.2)	17 (6.4)	
DSA (yes)	28 (10.4)	31 (11.6)	0.766
ABO incompatibility	6 (2.2)	6 (2.2)	1.000
eGFR 1 year post Τx (mL/min/1.73 m^2^)	53 (43–66)	56 (46–69)	0.103
Years of follow-up	8 (4–16)	13 (8–17)	<0.001
eGFR at follow-up (mL/min/1.73 m^2^)	50 (35–66)	39 (25–57)	<0.001
Rejection (yes)	46 (17.2)	32 (11.9)	0.103
Rejection type			0.256
● T-cell-mediated	38 (14.2)	26 (9.7)	
● antibody-mediated	6 (2.2)	4 (1.5)	
● mixed	2 (0.8)	2 (0.8)	
CMV infection	25 (9.3)	49 (18.3)	0.003
BKV infection	7 (2.6)	9 (3.4)	0.593

BMI: body mass index, Tx: transplantation, HBV: hepatitis B virus, HCV: hepatitis C virus, PRAs: panel reactive antibodies, DSA: donor specific antibody, HLA: human leukocyte antigen, eGFR: estimated glomerular filtration rate, CMV: cytomegalovirus.

**Table 2:** Immunosuppressive medication and regimens among kidney transplant recipients with malignancy and without malignancy.

	Kidney Transplant Recipients with Malignancy (Ν = 268)	Kidney Transplant Recipients Without Malignancy (Ν = 268)	*p*-Value
Induction treatment			
Anti-IL2Ra antibodies	39 (14.5)	35 (13)	0.618
ATG/OKT3	139 (51.9)	145 (54.1)	0.505
No induction	90 (33.6)	88 (32.8)	0.880
Maintenance treatment			
Steroids	231 (86.2)	221 (82)	0.282
Calcineurin inhibitor (CNI)	252 (94)	247 (92.3)	0.848
● cyclosporin A	141 (52.6)	130 (48.5)	0.764
● tacrolimus	111 (41.2)	114 (42.5)	0.910
Azathioprine (Aza)	51 (19)	49 (18.3)	0.874
Μycophenolate (MMF/MPA)	195 (72.8)	198 (73.8)	0.703
mTOR inhibitors	21 (7.8)	24 (9)	0.296
Antirejection treatment			
Steroid pulses	39 (14.5)	28 (10.4)	0.101
ATG/OKT3	10 (3.7)	8 (3)	0.617
Rituximab±plasma exchange	3 (6.7)	4 (7.5)	0.887
Immunosuppressive regimens			
ΜΜF/MPA+CNI±MP	184 (66.7)	184 (66.7)	0.897
Aza+CNI±MP	46 (17.1)	42 (15.7)	
MMF/MPA+mTORi±MP	8 (3)	13 (4.8)	
CNI+mTORi±MP	13 (4.8)	11 (4.1)	
CNI+MP	9 (3.4)	10 (3.7)	
Aza+MP	5 (1.9)	7 (2.6)	
Other	3 (1.1)	1 (0.4)	

Anti-IL2Ra Abs: Antibodies against receptor a of interleukin 2, ATG: antithymocyte globulin, OKT3: muromonab, MMF: mycophenolate mofetil, MPA: mycophenolate sodium, MP: methylprednisolone, mTORi: mammalian target of rapamycin inhibitor.

**Table 3:** Multivariate logistic regression investigating potential risk factors in association with malignancy among kidney transplant recipients.

	Odds Ratio (OR)	95% CI	*p*-Value
Immunosuppressive Regimen			
ΜΜF/MPA+CNI±MP (reference)			
Aza+CNI±MP	1.16	(0.66, 2.04)	0.593
MMF/MPA+mTORi±MP	0.62	(0.24, 1.60)	0.330
CNI+mTORi±MP	1.14	(0.52, 2.53)	0.623
CNI+MP	0.97	(0.36, 2.60)	0.958
Aza+MP	0.80	(0.23, 2.74)	0.724
Induction treatment			
No induction (reference)			
ATG/OKT3	1.01	(0.54, 1.81)	0.990
Anti-IL2Ra Abs	0.82	(0.48, 1.34)	0.405
Rejection	1.75	(1.04, 2.94)	0.034
eGFR 1 year post Τx (mL/min/1.73 m^2^)	0.99	(0.98, 1.01)	0.085
Dialysis vintage (months)	1.00	(0.99, 1.01)	0.890
Primary kidney disease			
unknown (reference)			
glomerulonephritis	2.23	(1.06, 4.67)	0.034
polycystic kidney disease	0.83	(0.49, 1.39)	0.478
hypoplastic kidneys/congenital nehropathy	0.78	(0.39, 1.55)	0.493
obstructive nephropathy/lithiasis	1.23	(0.78, 1.96)	0.358
diabetic nephropathy	0.69	(0.24, 1.91)	0.480
hypertensive nephropathy	0.78	(0.23, 2.67)	0.700

MMF: mycophenolate mofetil, MPA: mycophenolate sodium, CNI: calcineurin inhibitor, MP: methylprednisolone, Aza: azathioprine, mTORi: mammalian target of rapamycin inhibitor, ATG: antithymocyte globulin, OKT3: muromonab, Anti-IL2Ra Abs: Antibodies against receptor a of interleukin 2, eGFR: estimated glomerular filtration rate, Tx: transplantation.

## Data Availability

The original contributions presented in this study are included in the article. Further inquiries can be directed to the corresponding author.
